# Genome-wide identification and characterization of *HSP90* family gene in cotton and their potential role in salt stress tolerance

**DOI:** 10.3389/fpls.2025.1574604

**Published:** 2025-07-02

**Authors:** Zhao Yan Hao, Qian Feng, Xu Yan Man, Dong Qi Qi, Yu Shi Qing, Zhai Wei Yang, Mohssen Elbagory, Esam Sayed Kasem, Muhammad Yasir, Jiang Yu Rong

**Affiliations:** ^1^ College of Advanced Agricultural Science, Zhejiang Agriculture and Forestry University, Hangzhou, China; ^2^ Agricultural Extension Department, Tonglu County Agricultural Technology Extension Center, Hangzhou, China; ^3^ Jixian Honors College, Zhejiang Agriculture and Forestry University, Hangzhou, China; ^4^ Financial Management Office of Sixi Town People's Government, Taishun County, Wenzhou, Zhejiang, China; ^5^ Health Specialties, Basic Sciences and Applications Unit, Applied College, King Khalid University, Mohail Assir, Saudi Arabia; ^6^ Soil, Water and Environment Research Institute, Agriculture Research Center, Giza, Egypt; ^7^ College of Tea Science and Tea Culture, Zhejiang Agriculture and Forestry University, Hangzhou, China

**Keywords:** *Gossypium hirsutum*, *Hsp90*, salt stress, VIGS, cotton

## Abstract

Heat shock proteins 90 (HSP90s) are conserved molecular chaperones essential for cellular homeostasis under abiotic stress. While several studies have been reported to elucidate the role of HSP90s in heat stress responses in cotton, their involvement in salt stress remains underexplored. *Gossypium hirsutum* L. is highly susceptible to salt stress. The Current study investigated the evolutionary aspects, expression patterns, and functional validation of *HSP90* family members in cotton under salt stress. A comprehensive genomic analysis of *G. hirsutum*, *G. raimondii*, and *G. arboreum*, identified 56 *HSP90* genes which were classified into three distinct phylogenetic groups. Gene structure and motifs analysis revealed a conserved nature of *HSP90s* within each group. Additionally, *cis*-acting elements suggested the potential roles of *HSP90s* in biotic and abiotic stresses. The Ka/Ks ratio of all genes was < 1 suggesting negative and purifying selection pressure during molecular evolution. Expression analysis demonstrated the potential role of *HSP90* genes in salt tolerance. Notably, out of ten *HSP90* genes five genes exhibited highly differential expression under salt stress, as confirmed by qRT-PCR analysis. Moreover, virus-induced silencing of the two salt stress-responsive genes, *Ghir_D03G016230* and *Ghir_D02G013530*, which were upregulated under salt stress, resulted in a significant decrease in SOD, POD, and CAT, accompanied by a marked increase in MDA content in the salt-tolerant cotton cultivar. These findings provide novel insights into the functional roles of *HSP90s* in *G. hirsutum* under salt stress.

## Introduction

Plants have evolved intricate mechanisms to survive abiotic stresses, including heat and salinity. These include stress avoidance, osmotic adjustment, antioxidant defense, stress signaling, symbiotic association, and epigenetic regulation. Rather than working independently, these mechanisms function in a coordinated way to improve plant resilience. For example, stress avoidance strategies like stomata closure and dormancy alongside stress tolerance mechanisms such as osmolyte accumulation and antioxidant production synergistically mitigate the effects of abiotic stress (Bandurska, 2022). Symbiotic association with endophytic fungi improves stress tolerance by inducing physiological and molecular changes in host plants (Rodriguez et al., 2008). Stress signaling, involving hormones like salicylic acid and abscisic acid modulates stress-responsive genes leading to the activation of various tolerance mechanisms simultaneously (Khan et al., 2015; Sha Valli Khan et al., 2014; Yasir et al., 2019). Epigenetic regulation through DNA methylation, histone modifications, and small RNAs further fine-tune gene expressions. These coordinated mechanisms suggest shared regulatory pathways and cross-talk between stress responses.

Among various molecular players mediating stress responses, heat shock proteins (HSP90) are critical. The HSP90 supports stress signaling pathways and assists in the proper functioning of stress-induced proteins. Recent studies have unraveled the potential of *HSP90* family genes as targets for improving stress tolerance in crops, including cotton, which is susceptible to salinity. While heat shock protein (HSP) families are primarily involved in heat stress response, growing evidence suggests their involvement in salt tolerance as well. For instance, overexpression of *ZmHsf01* and *OsHsp20* in *Arabidopsis thaliana* enhanced tolerance to both heat and salt stress (Huang et al., 2022). Similarly, the maize gene *ZmHsf11* negatively regulates heat stress tolerance by modulating oxidative stress-related genes, which are also implicated in salt stress response (Qin et al., 2022). Moreover, genes like *OsRCI2–10* and *PGR5*, upregulated in salt-tolerant rice genotypes, are annotated for abiotic stress response, indicating their potential roles in both salt and heat tolerance (Razzaque et al., 2019). The Na+/H+ antiporter NHX1, known for salt tolerance by sequestering Na+ ions in vacuoles, also improves thermotolerance (Trono and Pecchioni, 2022). These findings highlight the existence of shared genetic networks and cross-tolerance mechanisms. When stimulated by various factors such as heat, plants produce highly conserved proteins known as heat shock proteins (HSPs) (Hsu et al., 2003; Queitsch et al., 2002; Robert, 2003). HSPs are well-characterized proteins that function as molecular chaperones, ensuring the proper folding and protection of cellular proteins (King & MacRae, 2015). HSPs have been identified across plants and animals (Xiong and Ishitani, 2006). These proteins have been categorized into several families based on their molecular weights, including the HSP100/ClpB family, HSP90 family, HSP70/DnaK family, chaperonin (HSP60/GroEL) family, and small heat-shock proteins (sHSP) family (Al-Whaibi, 2011; Caplan et al., 2003; Pratt and Toft, 2003; Wang et al., 2004).

The *HSP90* gene family was initially discovered in Drosophila with a single gene, *Hsp82*. HSP90 chaperones are expressed constitutively in most organisms under normal conditions, however their expression increases significantly under stress. HSP90, as a class of chaperones, is involved in DNA repair, substrate activation, and initial stress signaling (Lachowiec et al., 2014; Shinozaki et al., 2006; Zuehlke and Johnson, 2010). It functions to prevent protein aggregation and facilitates the refolding of inactive proteins in both normal and stress conditions (Picard, 2002). When plants are exposed to stress, the upregulated HSP90 interacts with non-proteinaceous substances, repairing deformed proteins (Mukhopadhyay et al., 2003). HSP90 is highly expressed in plant cells, constituting about 1-2% of total protein in the cytoplasm, and is characterized by conserved amino acid sequences (Prasinos et al., 2004). HSP90 consists of three domains: an N-terminal ATP-binding motif, a middle domain, and a C-terminal domain (CTD) responsible for HSP90 dimerization. This protein family acts as a mediator in plant abiotic stress signal pathways however, the underlying mechanisms remain unclear (Liu and Ekramoddoullah, 2006).

Although the HSP90 protein is well studied in heat tolerance in, however, due to the integrated mechanism of stress tolerance in plants we hypothesized that this protein family has a potential role in salt tolerance in upland cotton. Previous studies identified 9, 7, and 21 *HSP90* genes in *Oryza sativa*, *Arabidopsis thaliana*, and *Nicotiana tabacum*, respectively (Hu et al., 2009; Krishna and Gloor, 2001). To the best of our knowledge, no genome-wide study has yet explored the role of the HSP90 protein family in cotton under salt stress. Upland cotton is an important commercial crop that provides feed and food for animals and humans, and lint for the textile industry (Yasir et al., 2022). However, salt stress is one of the major challenges for sustainable cotton production. Therefore, breeding for salt tolerance is an important objective of cotton breeding. In this study, we conducted a genome-wide analysis of HSP90 family members, analyzing gene structures, evolutionary relationships, chromosomal locations, and conserved domains in detail using bioinformatics tools. Additionally, we investigated their expression patterns under salt stress through qRT-PCR and validated results through virus-induced gene silencing (VIGS), antioxidants scavenging enzymes (SOD, POD, CAT), and oxidative stress marker i.e., malondialdehyde (MDA). These results provide a foundation for understanding the genomic organization and functional role of the HSP90 gene family in *G. hirsutum*.

## Materials and methods

### Plant materials and stress treatments

Salt-tolerant cotton lines HNZ2019–2520 and salt-sensitive line HNZ2019-2521 were used for gene expression experiments. The seeds were surface sterilized with 10% sodium hypochlorite and three times rinsed with distilled water followed by planting in mixed soil (vermiculite: humus = 1:1) saturated with water, in trays in a greenhouse at 22°C with a 16 h/8 h (light/dark) photoperiod until complete unfolding of cotyledonary leaves. Seedlings were then shifted to hydroponic boxes and divided into two groups for control and salt treatment, each having three replications. Salt treatment of 300 mM NaCl (validated to cause significant stress in a pilot experiment) at two true leaf stages. Untreated control plants were grown normally. Samples were taken at 0, 3, 12, 24, and 48, hours post-treatment with three biological and three technical repeats. Leaf, stem, and root tissues were sampled to determine tissue-specific analyses. After collection, all samples were quickly frozen in liquid nitrogen and stored at −80°C for RNA isolation and downstream analysis like qRT-PCR, VIGS, and biochemical analysis. RNA-seq expression data of *G. hirsutum* accession TM-1 was retrieved from NCBI-Bioproject PRJNA490626 (Hu et al., 2019).

### Identification of HSP90 genes

The *Arabidopsis thaliana* HSP90 protein sequences were retrieved from the TAIR databases (http://www.arabidopsis.org/) (Krishna and Gloor, 2001). The *Oryza sativa* and *Triticum aestivum* protein sequences were downloaded from NCBI (https://www.ncbi.nlm.nih.gov/) and *G. hirsutum _*HAU-AD1_v1.1 (TM-1), *G. arboreum* (A2) ‘SXY1’ genome CRI-updated_v1, and *G. raimondii* (D5) genome JGI_v2_a2.1 from cotton functional genomics database website (https://cottonfgd.net/). The Pfam (http://pfam.sanger.ac.uk/search) and SMART (http://smart.embl-heidelberg.de/) databases were employed to confirm each predicted HSP90 protein (Finn et al., 2015). Additional characteristics of HSP90 proteins, including amino acid (aa) or protein length and molecular weight (kDa), isoelectric points (pIs), grand average of hydropathy, and charge were analyzed using the Cotton Functional Genomic Database (http://www.cottonfgd.org/).

### Phylogenetic analysis

Multiple sequence alignment of HSP90 was performed using ClustalW (Larkin et al., 2007). The MEGA 7.0 software was employed to construct an unrooted phylogenetic tree by the neighbor-joining (NJ) method (Kumar et al., 2016) with 1000 bootstraps for statistical reliability.

### Chromosomal mapping of *HSP90* family genes

The genomic locations of all *HSP90* genes were obtained from the gff3 files of three cotton species available on the Cotton Functional Genomic Database (http://www.cottonfgd.org/). The TBtools software was employed to assess the chromosomal distribution and positions of *HSP90* genes (Chen et al., 2020).

### Gene structure motif analysis and sub-cellular localization of HSP90 family members

A diagrammatic sketch of the *HSP90* gene structure was constructed using the Gene Structure Display Server (GSDS) (http://gsds.cbi.pku.edu.cn) (Hu et al., 2015) based on the alignment of the CDS with their corresponding genomic DNA sequences. The conserved motifs of the full length of HSP90 family proteins were analyzed using the online MEME tool (Multiple Expectation Maximization for Motif Elicitation, http://memesuite.org/tools/meme) (Bailey et al., 2006). The maximum motif search value was set at 10. Sub-cellular localization of proteins was determined using online bioinformatics tools Cell-Ploc (Chou and Shen, 2008), WoLF PSORT (Horton et al., 2007), and LocTree3 (Goldberg et al., 2014) with their protein sequences.

### Quantitative RT-PCR expression analysis of HSP90 genes

Plant total RNA was extracted by the Total RNA Extraction Kit (R1200) (Beijing Solarbio Science & Technology Co., Ltd., Beijing, China). PrimeScript™ II 1st Strand cDNA Synthesis Kit (TAKARA, Dalian, China) was used for reverse transcription to obtain the first-strand cDNA for transcriptomic and RT qPCR analysis. The expression heatmap was visualized by TBtools. The fluorescent quantitative Taq Pro Universal SYBR qPCR Master Mix (Q712- 02) (Vazyme Biotech Co., Ltd, Nanjing China) kit was employed for the qRT-PCR reactions. The data were calculated according to the 2−^ΔΔCT^ method (Livak and Schmittgen, 2001). According to the candidate gene sequence, a relatively specific primer for real-time fluorescence quantitative PCR was designed by Oligo 7 software **Supplementary Table S1**, and the amplification product was 150–300 bp (Rychlik 2007; Livak and Schmittgen, 2001).

### Virus-induced gene silencing and stress treatment

The plasmid TRV2 (pYL156) and TRV1 (pYL192) were employed for VIGS (**Supplementary Figure 1**). The VIGS sequence was obtained using the SGN VIGS Tool (http://vigs.solgenomics.net/) and cloned into the TRV2 vector using the T4 DNA ligation cloning method. The lengths of the silencing fragments for *Ghir_D03G016230* and *Ghir_D02G013530* were 212 and 243 bp, respectively. The TRV-VIGS constructs were also transformed into *A. tumefaciens* strain GV3101. Then, strain GV3101, containing the different constructs of gene of interest, was infiltrated into 10-day-old cotton seedlings with fully expanded cotyledonary leaves according to the method described by Gao et al. (2011) *TRV2:CLA1* (CLOROPLASTOS ALTERADOS 1) was utilized as the positive control and the empty vector TRV2:00 was the negative control (Gao et al., 2011). When the plants grew up to 2~3 true leaves stage they were shifted to hydroponic boxes. The primers list has been given in **Supplementary Table S2**. Two weeks after infection, the albino leaf phenotype appeared in plants transformed with the positive control gene CLA1. Subsequently, cotton plants containing TRV2: *Ghir_D03G016230*, TRV2: *Ghir_D02G013530*, and the negative control TRV2:00 were exposed to salt treatments at two true leaf stage with the same volume of water or salt solution (300 mM/L NaCl) (**Supplementary Figure 2**). The plants were observed and photographed during the treatment. Furthermore, the effect of gene silencing on SOD, POD, CAT, and MDA was observed.

### Measurement of physiological indices

To determine the physiological indices variations, the candidate genes-silenced cotton leaves of the salt-tolerant cultivar were used to observe the activities of SOD, POD, CAT, and concentration of MDA after salt treatments. The SOD activity, CAT activity, and POD activities were detected according to the previous reports (Alici and Arabaci, 2016; Ullah et al., 2018). The MDA concentration was determined using thiobarbituric acid method (Schmedes and Hølmer, 1989). The absorbance was measured using a UV-2550 UV-vis spectrophotometer (SHIMADZU). Leaf relative water content and chlorophyl contents were measured as described in previous studies (Magwanga et al., 2019). All the samples were replicated three times.

## Results

### The assessment of salt tolerant and salt sensitive genotypes

**Figure 1:**
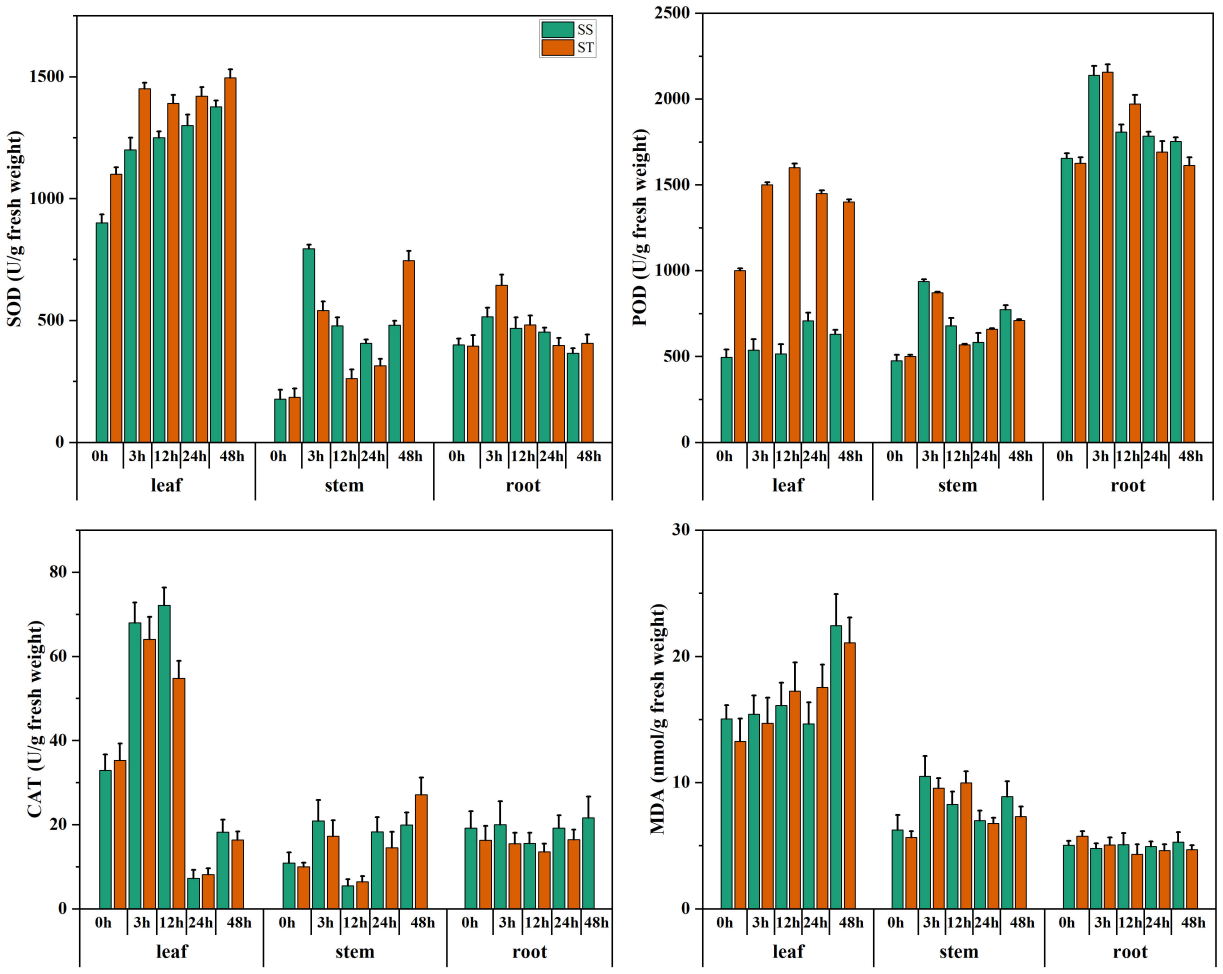
The assessment of salt tolerant and sensitive genotypes. The expression of SOD, POD, CAT and MDA of salt tolerant (ST) HNZ2019-2520, and salt sensitive (SS) HNZ2019-2521 genotypes. The red bar graph represents salt tolerant genotype whereas, green salt sensitive. Error bars represent the standard deviation of the triplicated data.

The salt-tolerant and salt-sensitive genotypes were subjected to salt stress. The biochemical outcomes of SOD, POD, CAT, and MDA from different tissues such as leaf, root, and stem revealed that activities of SOD, POD, and CAT were significantly high in salt tolerant genotype however, the MDA expression was low in salt-tolerant and vice versa as shown in **Figure 1**. These results corroborated the findings of HNZ2019–2520 as salt tolerant genotype, which was further used for downstream experiments and validation results.

### Genome-wide identification of HSP90 gene family members

**Figure 2:**
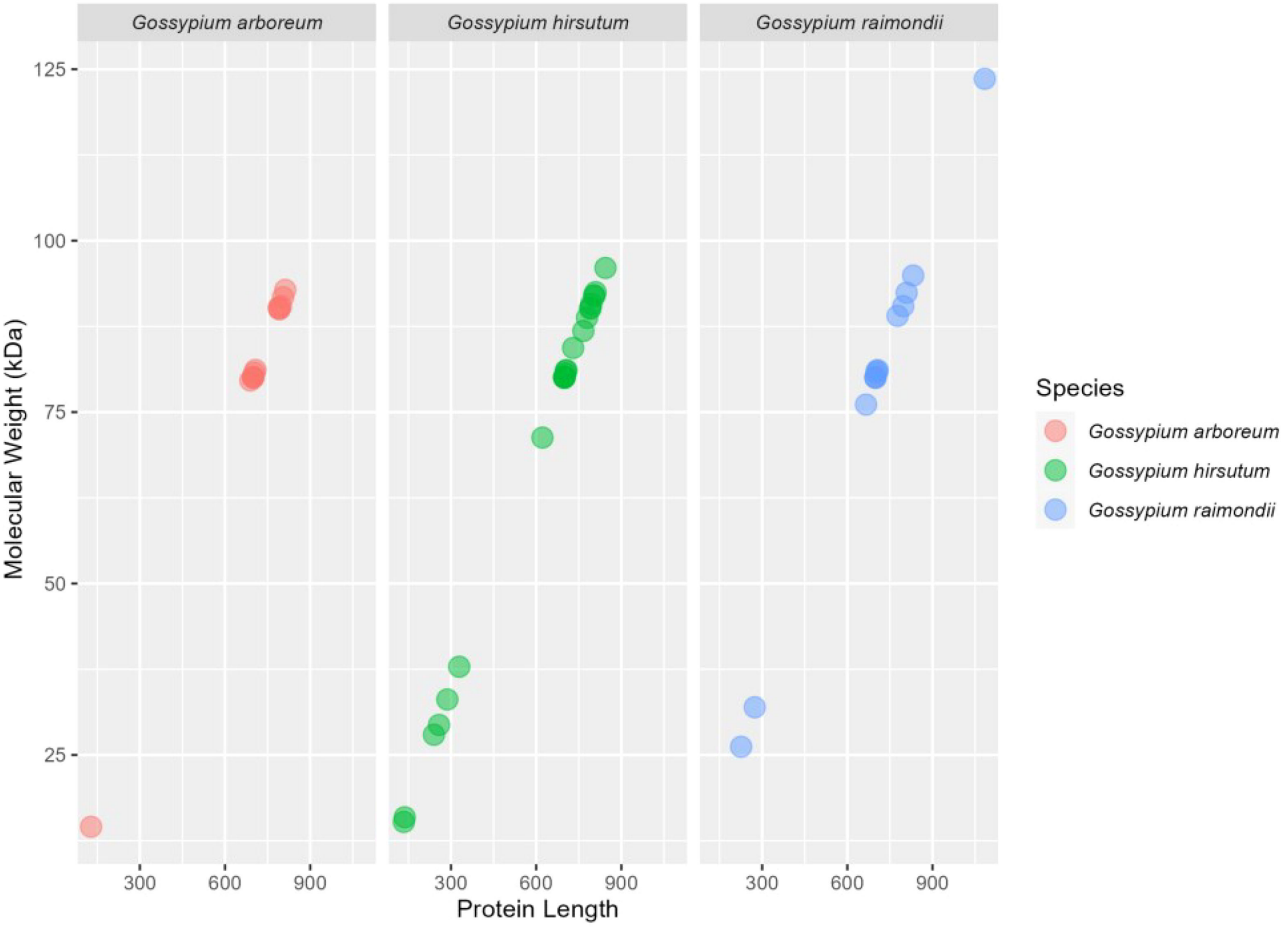
The plot depicts the estimation of the amino acids length on X-axis and molecular weight of HSP90 proteins on Y-axis.

A total of 28 *G. hirsutum*, 14 *G. arboreum*, and 14 *G. raimondii* genes were identified. The genomic, CDS, and protein features of the *HSP90* genes of *G. hirsutum* were then identified, such as genomic length (bp), protein length (aa), CDS length (bp), locus ID with corresponding chromosome number, strand polarity, start and end points, predicted isoelectric points (PI), predicted masses, and protein molecular weights (MW), and charges as shown in **Tables 1**, **2**. A comparative visualization of the protein length and molecular weight of HSP90 proteins has been shown in **Figure 2**. Retrieving the information of *HSP90* genes revealed that *Ga05G1968*, which was detected on chromosome chr05 in *G. arboreum*, coded the smallest protein of 128 amino acids (aa), with a molecular weight of 14.527 kDa. Meanwhile, *Gorai.010G003000*, identified on chromosome chr10 in *G*. *raimondii*, coded the largest protein among all HSP90 members in three *Gossypium* species, having 1084 aa, with a molecular weight of 123.601 kDa as shown in **Figure 2** and **Table 2**. The isoelectric point (PI) of the *HSP90* genes ranged from 4.423 (*Ghir_A05G018960*) to 9.324 (*Ghir_D11G023100*). The theoretical isoelectric point of all the proteins except three proteins was > 7. Whereas, the grand average of hydropathy of all *HSP90* proteins was negative. Moreover, in-silico analysis using Cell-Ploc, WoLF PSORT, and LocTree3 revealed that the highest number of genes were localized in the cytoplasm 26, 29, and 43 respectively, with the LocTree3 predicting the highest as given in **Supplementary Table S4**. Cell-Ploc and WoLF PSORT were significantly in agreement in terms of gene localization in the cytoplasm. The endoplasmic reticulum contained 14, 7, and 13 genes respectively, with WoLF PSORT predicting the most. The results of Cell-Ploc and LocTree3 were in compliance in terms of HSP90s localization to the endoplasmic reticulum.

**Table 1:** Physichochemical properties of three cotton species.

Gene ID	Protein Length (aa)	Molecular Weight (kDa)	Charge	Isoelectric Point	Grand Average of Hydropathy
*Ghir_A01G008350*	699	80.066	-27.5	4.726	-0.572
*Ghir_A01G009870*	794	90.742	-30.5	4.684	-0.689
*Ghir_A03G002790*	329	37.867	-8	5.042	-0.513
*Ghir_A03G002800*	137	15.92	-1	5.838	-0.573
*Ghir_A03G012060*	779	88.764	-27.5	4.682	-0.557
*Ghir_A05G018960*	134	15.239	-7	4.423	-0.457
*Ghir_A06G000260*	805	91.916	-37	4.605	-0.713
*Ghir_A07G020520*	622	71.284	-16.5	4.848	-0.633
*Ghir_A08G003090*	699	80.054	-27.5	4.726	-0.579
*Ghir_A08G003100*	700	80.139	-28.5	4.704	-0.571
*Ghir_A08G026410*	258	29.383	-15.5	4.466	-0.531
*Ghir_A12G026820*	703	80.643	-26.5	4.718	-0.605
*Ghir_A13G009660*	766	86.834	-35.5	4.519	-0.518
*Ghir_A13G013590*	699	80.143	-26.5	4.748	-0.591
*Ghir_D01G008810*	699	80.022	-27.5	4.726	-0.572
*Ghir_D01G010630*	809	92.49	-31	4.693	-0.688
*Ghir_D02G013530*	790	90.159	-33.5	4.589	-0.569
*Ghir_D03G016230*	707	81.116	-23.5	4.815	-0.617
*Ghir_D06G000070*	804	92.04	-34	4.654	-0.718
*Ghir_D06G016230*	240	27.947	-1	5.979	-0.587
*Ghir_D07G020620*	844	96.022	-8	5.443	-0.527
*Ghir_D08G003190*	699	80.022	-27.5	4.728	-0.584
*Ghir_D08G003210*	699	80.048	-27.5	4.729	-0.576
*Ghir_D08G012920*	704	81.051	-25	4.772	-0.595
*Ghir_D11G023100*	287	33.097	11.5	9.324	-0.122
*Ghir_D12G026910*	730	84.354	-2	6.219	-0.525
*Ghir_D13G009090*	791	90.098	-32	4.605	-0.428
*Ghir_D13G014300*	699	80.125	-27.5	4.725	-0.589
*Ga01G0999*	699	80.066	-27.5	4.726	-0.572
*Ga01G1189*	812	92.816	-33	4.66	-0.695
*Ga01G2599*	707	81.145	-27.5	4.728	-0.609
*Ga02G1769*	699	80.001	-28.5	4.704	-0.57
*Ga02G1770*	699	80.068	-27.5	4.726	-0.58
*Ga03G1468*	790	90.034	-32.5	4.602	-0.56
*Ga05G1968*	128	14.527	-9	4.428	-0.663
*Ga06G0030*	789	90.293	-40	4.549	-0.729
*Ga06G0031*	804	91.746	-36.5	4.599	-0.702
*Ga07G2203*	797	90.453	-15.5	4.995	-0.577
*Ga08G1356*	689	79.615	-23	4.815	-0.628
*Ga12G0240*	703	80.742	-27.5	4.701	-0.613
*Ga13G1166*	792	90.016	-33.5	4.577	-0.564
*Ga13G1641*	699	80.139	-26.5	4.748	-0.587
*Gorai.001G220600*	797	90.448	-15.5	4.995	-0.569
*Gorai.002G103000*	699	80.007	-28.5	4.707	-0.566
*Gorai.002G122800*	809	92.407	-32	4.674	-0.684
*Gorai.003G155600*	707	81.117	-24.5	4.792	-0.617
*Gorai.004G033900*	699	80.02	-27.5	4.728	-0.576
*Gorai.004G034000*	666	76.096	-24.5	4.765	-0.561
*Gorai.004G138600*	704	81.025	-24	4.794	-0.591
*Gorai.005G148100*	832	94.919	-34.5	4.578	-0.47
*Gorai.007G238900*	274	31.959	4.5	8.557	-0.398
*Gorai.008G274600*	703	80.744	-27	4.724	-0.613
*Gorai.010G003000*	1,084	123.601	-34	4.816	-0.666
*Gorai.010G173700*	226	26.195	3	8.415	-0.522
*Gorai.013G098300*	777	89.036	-32	4.639	-0.506
*Gorai.013G150300*	699	80.125	-27.5	4.725	-0.589

**Table 2:** Genomic features of three cotton species.

Gene ID	Chromosome	Start	End	Strand	Length (bp)
*Ghir_A01G008350*	A01	14,553,402	14,556,889	–	3,488
*Ghir_A01G009870*	A01	21,024,434	21,028,720	+	4,287
*Ghir_A03G002790*	A03	4,241,366	4,243,296	+	1,931
*Ghir_A03G002800*	A03	4,243,323	4,243,736	+	414
*Ghir_A03G012060*	A03	70,117,279	70,123,061	+	5,783
*Ghir_A05G018960*	A05	17,997,552	17,998,809	+	1,258
*Ghir_A06G000260*	A06	168,499	179,965	+	11,467
*Ghir_A07G020520*	A07	89,766,421	89,770,702	–	4,282
*Ghir_A08G003090*	A08	3,069,702	3,072,964	–	3,263
*Ghir_A08G003100*	A08	3,101,817	3,105,495	–	3,679
*Ghir_A08G026410*	Scaffold1354	10,888	12,251	–	1,364
*Ghir_A12G026820*	A12	105,567,937	105,571,751	–	3,815
*Ghir_A13G009660*	A13	50,901,086	50,906,257	+	5,172
*Ghir_A13G013590*	A13	88,404,614	88,408,124	+	3,511
*Ghir_D01G008810*	D01	12,233,303	12,236,902	–	3,600
*Ghir_D01G010630*	D01	16,985,080	16,989,632	+	4,553
*Ghir_D02G013530*	D02	45,437,332	45,444,255	+	6,924
*Ghir_D03G016230*	D03	48,953,760	48,957,307	–	3,548
*Ghir_D06G000070*	D06	42,078	53,776	+	11,699
*Ghir_D06G016230*	D06	54,670,770	54,673,493	–	2,724
*Ghir_D07G020620*	D07	51,989,192	51,994,307	–	5,116
*Ghir_D08G003190*	D08	2,955,211	2,962,627	–	7,417
*Ghir_D08G003210*	D08	2,974,607	2,978,282	–	3,676
*Ghir_D08G012920*	D08	44,530,614	44,533,841	–	3,228
*Ghir_D11G023100*	D11	34,873,214	34,875,498	–	2,285
*Ghir_D12G026910*	D12	60,716,055	60,719,148	–	3,094
*Ghir_D13G009090*	D13	18,346,522	18,351,714	+	5,193
*Ghir_D13G014300*	D13	46,081,670	46,084,965	+	3,296
*Ga01G0999*	Chr01	15,324,156	15,327,045	–	2,890
*Ga01G1189*	Chr01	22,951,441	22,955,605	+	4,165
*Ga01G2599*	Chr01	110,018,507	110,021,550	+	3,044
*Ga02G1769*	Chr02	98,686,251	98,689,179	+	2,929
*Ga02G1770*	Chr02	98,720,919	98,723,567	+	2,649
*Ga03G1468*	Chr03	94,539,619	94,545,399	+	5,781
*Ga05G1968*	Chr05	17,861,689	17,862,394	+	706
*Ga06G0030*	Chr06	171,955	176,076	+	4,122
*Ga06G0031*	Chr06	178,338	182,462	+	4,125
*Ga07G2203*	Chr07	89,108,505	89,113,619	–	5,115
*Ga08G1356*	Chr08	92,103,514	92,111,427	–	7,914
*Ga12G0240*	Chr12	1,914,986	1,917,873	+	2,888
*Ga13G1166*	Chr13	59,371,166	59,376,339	+	5,174
*Ga13G1641*	Chr13	102,610,798	102,613,481	+	2,684
*Gorai.001G220600*	Chr01	44,749,309	44,754,987	–	5,679
*Gorai.002G103000*	Chr02	13,118,219	13,121,608	–	3,390
*Gorai.002G122800*	Chr02	17,649,893	17,654,527	+	4,635
*Gorai.003G155600*	Chr03	42,463,639	42,467,252	–	3,614
*Gorai.004G033900*	Chr04	2,773,498	2,776,641	–	3,144
*Gorai.004G034000*	Chr04	2,786,127	2,789,058	–	2,932
*Gorai.004G138600*	Chr04	38,870,293	38,873,451	–	3,159
*Gorai.005G148100*	Chr05	40,579,037	40,585,408	+	6,372
*Gorai.007G238900*	Chr07	32,618,140	32,619,777	–	1,638
*Gorai.008G274600*	Chr08	55,290,618	55,294,359	–	3,742
*Gorai.010G003000*	Chr10	124,875	135,837	+	10,963
*Gorai.010G173700*	Chr10	50,606,514	50,609,166	–	2,653
*Gorai.013G098300*	Chr13	17,479,456	17,484,611	+	5,156
*Gorai.013G150300*	Chr13	41,037,208	41,040,239	+	3,032

### Evolutionary relationship and classification of HSP90 family genes

**Figure 3:**
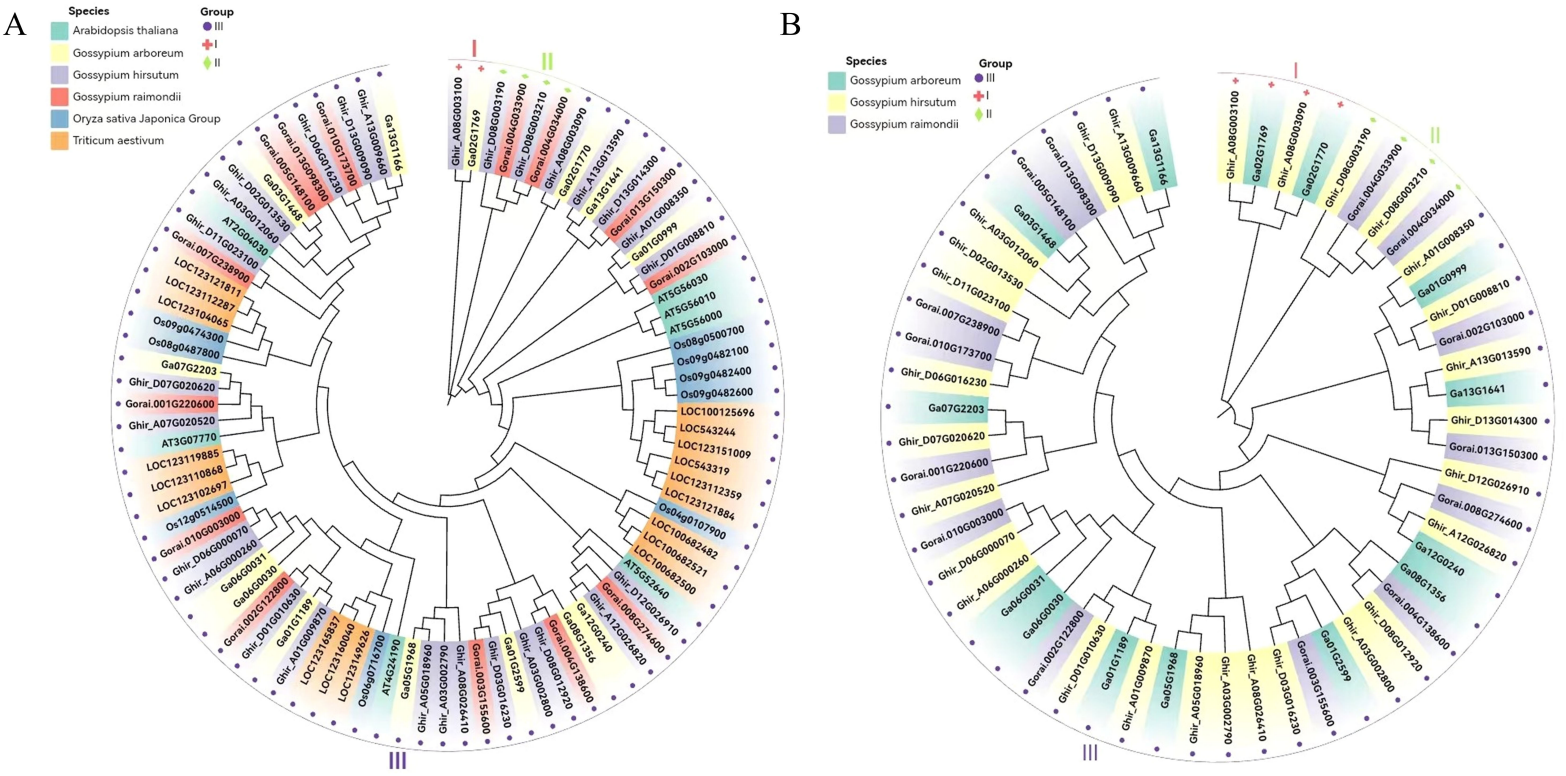
Phylogenetic analysis. **(A)** Neighbor-joining phylogenetic tree of 56 *HSP90* genes of the three cotton species, model plant *Arabidopsis thaliana, Oryza sativa*, and *Triticum aestivum.* **(B)** Phylogenetic tree of three cotton species.

To analyze the evolutionary relationships of the *HSP90* genes, we constructed a neighbor-joining (NJ) phylogenetic tree based on multiple sequence alignments of 56 HSP90 proteins of *Gossypium* species (*G. hirsutum* (28), *G. arboreum* (14), and *G. raimondii* (14), 7 proteins of *A. thaliana*, 9 of *Oryza sativa*, and 18 of *Triticum aestivum* as shown in **Figure 3A**. To get further insights into the evolutionary relationship among the three cotton species under study, another phylogenetic tree was constructed as shown in **Figure 3B**. Both the phylogenetic trees were divided into three subgroups (**Figures 3A, B**). The *Arabidopsis thaliana*, *Oryza sativa*, and *Triticum aestivum* were exclusively present in clade III with *Gossypium* species whereas clade I and II contained only cotton genes. *Ghir_A08G003100* and *Ga02G1769* were present in clade I, whereas *Ghir_D08G03190, Ghir_DO8G003210* of *G. hirsutum, Gorai.004G033900* and *Gorai.004G034000* of *G. raimondii* were present in clade II **Figure 3A**. A similar pattern was observed in the phylogenetic tree of cotton species as shown in **Figure 3B**.

### Chromosomal mapping

**Figure 4:**
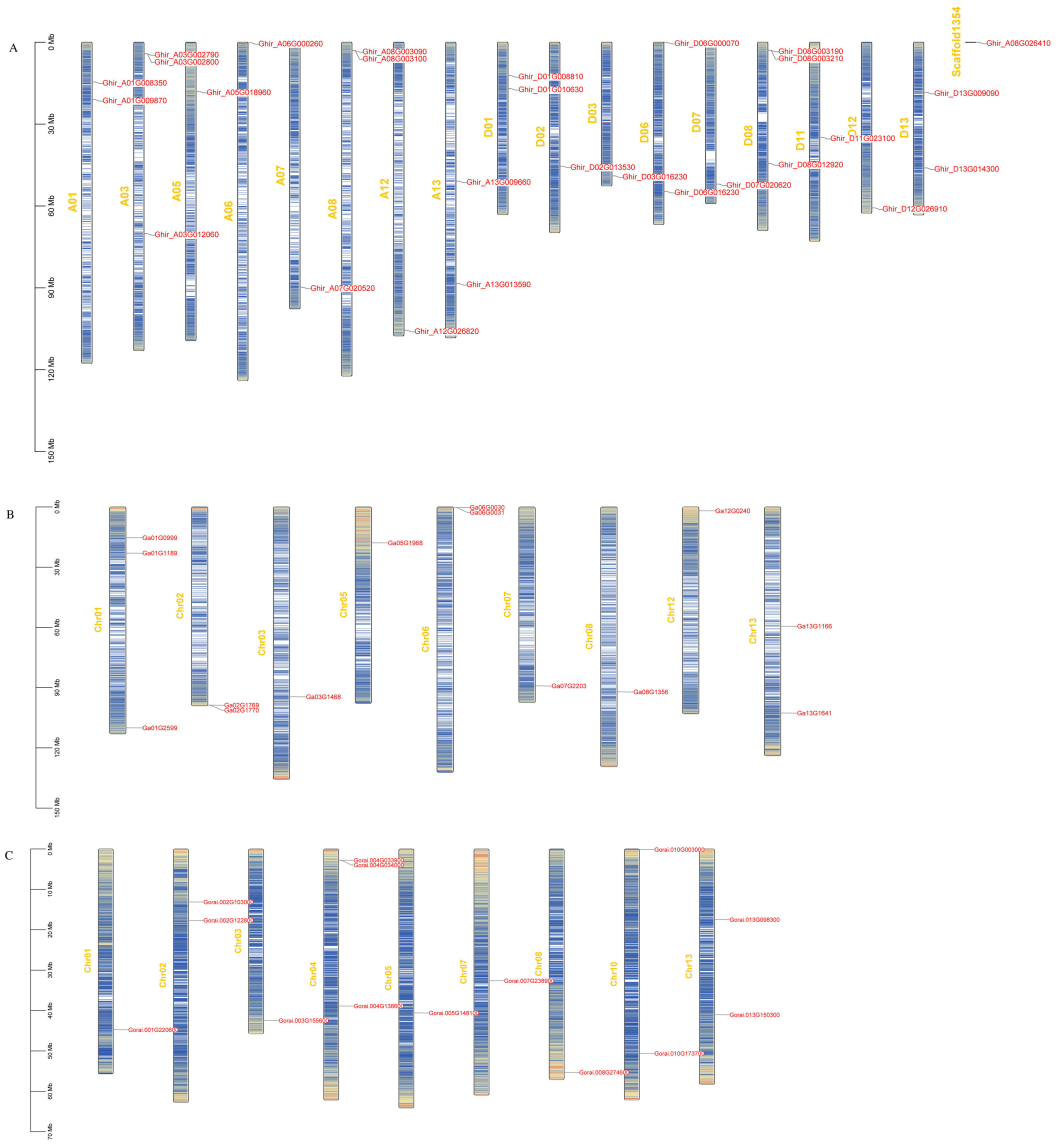
**(A-C)** Chromosomal mapping of *HSP90* genes on chromosomes. The name of the chromosomes are on the left whereas, gene IDs are placed on the right **(A)** *G. hirsutum*, **(B)** *G. arboreum*, **(C)** *G. raimondii*.

To determine the division of *HSP90* genes on different chromosomes of cotton, we constructed the chromosomal map with information taken from three cotton genomes as demonstrated in **Figure 4**. The results showed that most of the 56 genes were scattered irregularly across 13 chromosomes, while one gene *Ghir_A08G026410* was located on a scaffold. *G. hirsutum* was attributed with 13 genes distributed on A-subgenome whereas 14 genes were located on D-subgenome. Moreover, chromosomes *A03* and *D08* were found to have the highest number of *HSP90* genes, having 3 genes on each chromosome, followed by chromosomes *A01, A08, A13, D01, D06*, and *D13*, with 2 genes each; the lowest number of genes 1 was found on chromosomes *A05, A06, A07, A12, D02, D03, D07, D11* and *D12*. In *G. arboreum*, chromosome 01 had the highest number of gene 3, followed by 2 genes on chr 02, chr06, and chr13 the lowest number of gene 1 was found on chromosomes 03, chr05, chr07, chr08, and chr12. In *G. raimondii*, the highest number of genes were found on chromosome 04, with 3 genes, and the lowest number of genes only one *HSP90* member was found on chromosomes chr01, chr03, chr05, chr07, and chr08. Moreover, to explore the selection pressure we calculated the Ka, Ks, and Ka/Ks values of identified genes. The ka/Ks > demonstrates a positive selection, whereas Ka/Ks = 1 denotes neutral selection and Ka/Ks <1 corresponds to the negative selection pressure. In the current study, the Ka/Ks values of all the *HSP90* genes were < 1, suggesting that these gene pairs evolved under negative selection pressure in upland cotton as given in **Supplementary Table S3**.

### Gene structure, conserved motifs, and *Cis*-regulatory elements

**Figure 5:**
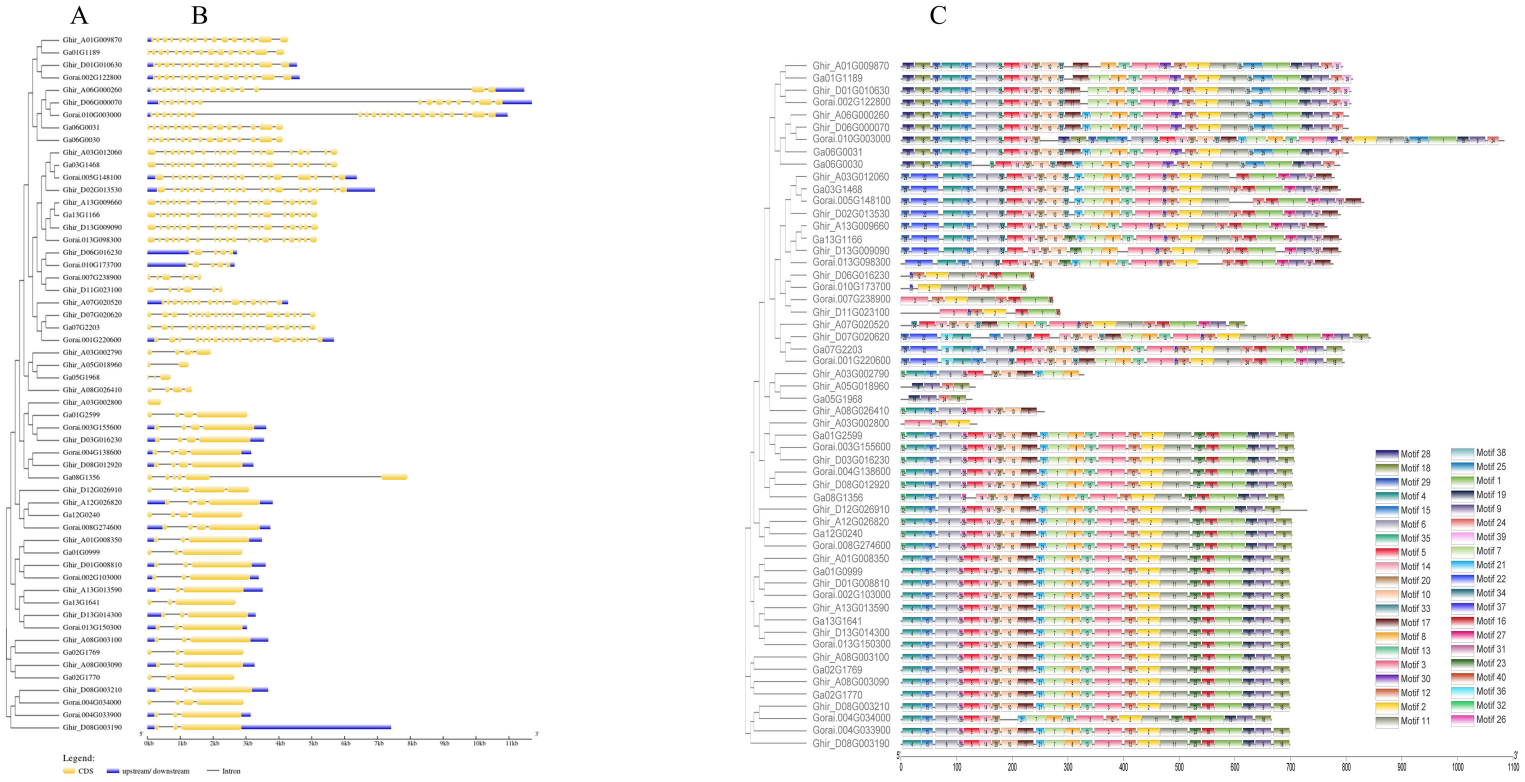
The phylogenetic tree, gene structure and conserved motif of HSP90 genes in three cotton species. **(A)** Multiple sequence alignment of candidate genes **(B)** Gene structure of *HSP90s*, the exons are represented by yellow boxes, intron by grey lines and upstream/downstream regions by purple boxes. **(C)** Conserved motifs of HSP90s. the motifs are displayed in different colors.

**Figure 6:**
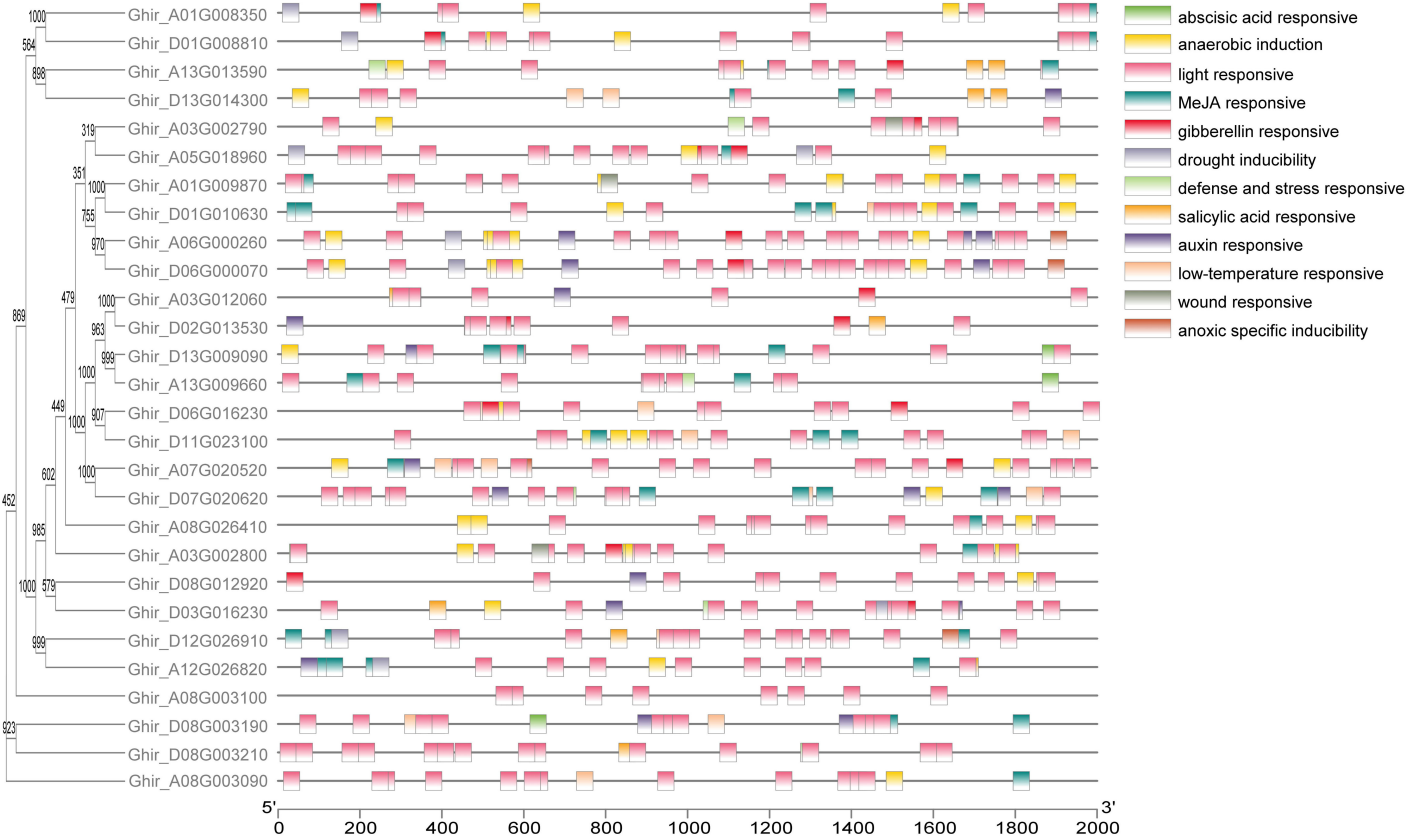
*Cis*-acting elements on promoter regions of *HSP90* genes in *G.hirsutum.* The evolutionary tree and cis-acting elements of *HSP90s*. Different colors represent *cis*-acting elements mediating stress tolerance pathway.

To determine the gene structures and evolutionary tree of *HSP90* genes, the intron-exon structures of 56 cotton genes were analyzed using the TBtools software, as shown in **Figures 5A, B**. The analysis revealed that one gene *Ghir_A03G002800* had only one exon, while the gene *Gorai.010G003000* had the highest number of exons i.e., 22. Forty motifs were identified using (http://meme-suite.org/. Motifs 1 and 2 were found most conserved out of all 40 motifs as shown in **Figure 5C**. The *cis*-acting elements in the promoter region play important roles in the plant responses to stress, and, participate in the responses to drought, ABA, and other stresses (Yazaki and Kikuchi, 2005; Nakashima and Yamaguchi-Shinozaki, 2013). The twelve most abundant *cis*-acting elements detected in HSP90s were the abscisic acid-responsive, anaerobic induction, light responsive, Methyl jasmonate responsive, gibberellin responsive, drought inducibility, defense and stress-responsive, salicylic acid-responsive, auxin-responsive, low temperature responsive, wound responsive and anoxic specific inducibility. Light responsive, Meja responsive, and anaerobic induction has the largest number of *cis*-regulatory elements followed by low temperature responsive and auxin-responsive as shown in **Figure 6**.

### Transcriptome profiling and qRT-PCR analysis

**Figure 7:**
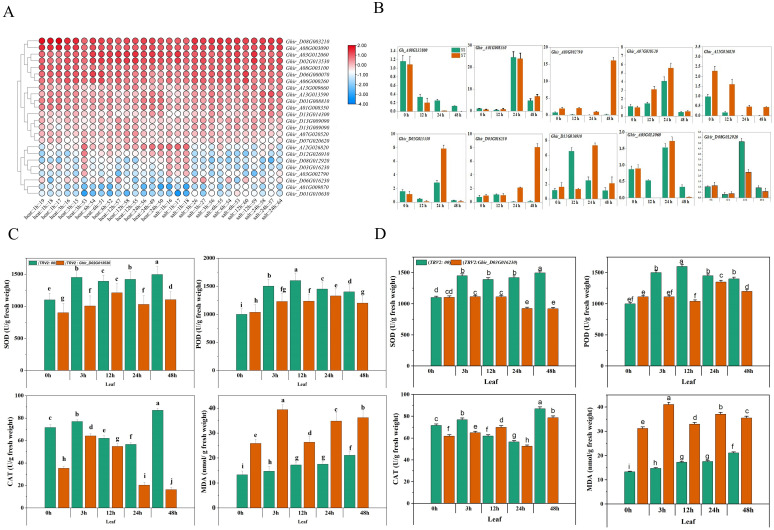
RNA expression profiling and qRT-PCR of *HSP90* genes of *G.hirsutum.* **(A)** Heatmap showing expression of *HSP9*0 genes under heat and salt stress at different time intervals. **(B)** qRT-PCR of ten genes under salt stress **(C)** Comparative expression of SOD, POD, CAT and MDA in leaf tissues of *Ghir_D03G016530* and **(D)** *Ghir_D02G013230* silenced plants of *G.hirsutum*. Different letters represent significant difference at p < 0.05.

Publicly available transcriptomic data of Texas Marker-1 (TM-1) was employed to unravel the differential expression of *HSP90* genes. Differential expression quantitative analysis FPKM (Fragments Per Kilobase of transcript per Million fragments mapped) refers to the number of fragments per thousand bases of a gene per million fragments, which is related to the length and expression level of transcripts, and can usually be used as transcript frequency or gene expression level (Zhao et al., 2021). The cluster analysis and heat-map visualization based on the level of expression of transcripts divided *HSP90* genes into three groups A, B, and C. In A group which includes 28 genes, 6 were differentially expressed at 1, 3, 6, 12, and 24 hours after heat and salt stress application (**Figure 7A**). Notably, *Ghir_D08G003210* and *Ghir_A08G003090* depicted early differential expression following 1-hour heat treatment. Moreover, *Ghir_A03G012060* and *Ghir_D02G013530* were highly expressed after 6 and 12 hours of heat stress. Interestingly, *Ghir_D08G003210* and *Ghir_A08G003090* were also expressed during salt stress at 3, 6, 12, and 24 hours of salt stress. In the B group, *Ghir_A13G013590* revealed high expression after 24-hour salt stress. In the C group, *Ghir_A12G026820* exhibited high expression under 1h salt and 24h heat stress but the remaining members in this group showed significantly low expression. To validate these findings, ten genes were selected for qRT-PCR analysis. Among them, *Ghir_A03G002790* was significantly upregulated at 48h salt treatment in salt-tolerant cultivar as compared to salt-sensitive cultivar (**Figure 7B**). Similarly, *Ghir_D03G016230*, *Ghir_A07G020520*, *Ghir_D02G013530*, and *Ghir_D12G026910* depicted significant differential expression between salt-tolerant and salt-sensitive cotton cultivars. Finally, based on these qRT-PCR results, we selected two differentially expressed genes *Ghir_D03G016230* and *Ghir_D02G013530* for functional validation.

### Effect of gene silencing on ROS scavenging enzymes (SOD, POD, CAT), and lipid peroxidation marker MDA

**Figure 8:**
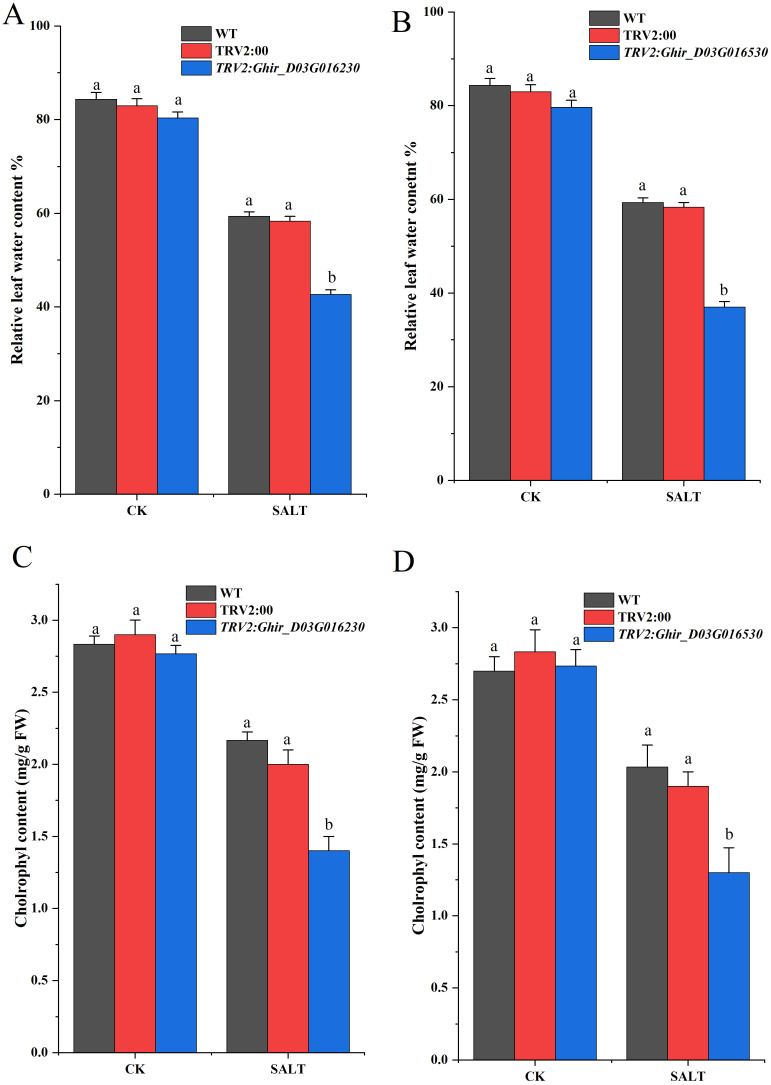
Quantitative determination of chlorophyl and leaf water contents. **(A,B)** Relative leaf water content of wild-type, control and *Ghir_D02G013230* and *Ghir_D03G016530* silenced plants **(C,D)** chlorophyl contents of wild-type, control and *Ghir_D02G013230* and *Ghir_D03G016530* silenced plants after 8-days of salt stress exposure. Letters depict significant differences (two-tailed, *p* < 0.01). each experiment was conducted in triplicate.

Reactive oxygen species (ROS) scavenging enzymes such as SOD (Superoxide Dismutase) POD (Peroxidase) and, CAT (Catalase) play an imperative role in the plant’s defense mechanism against oxidative stress (Fujita and Hasanuzzaman, 2022). The silencing of *Ghir_D02G013530* and *Ghir_D03G016230* exhibited a significant difference in the expression of the scavenging enzymes and lipid peroxidation marker under salt stress at different time intervals. **Figures 7C, D** exhibits the contents of SOD, POD, CAT, and MDA in cotton seedlings subjected to the silencing of upregulated genes. The silencing of *Ghir_D02G013530* exhibited an overall decrease of 23.43% in SOD content, 13.29% in POD content, and 49.53% in CAT contents of leaf tissue under salt stress. Moreover, a 93.71% increase in MDA content was observed. The lowest SOD, POD, and CAT contents -30.65%, -22.89%, and -85.61%, were found at 3h, 12h, and 24h time intervals respectively. Likewise, the highest MDA content 167.47% increase was found at a 3h time interval. Similarly, the silencing of *Ghir_D03G016230* resulted in an average decrease of 24.50% in SOD expression, 16.32% in POD expression, and 7.30% in CAT expression in leaf tissue of the cotton seedlings under salt stress. Whereas, an overall 112.22% increase in MDA content was observed in *Ghir_D03G016230* silenced plants, moreover highest differences of SOD, POD, and CAT contents -38.47%, -34.94%, and -15.45% were found at 48h, 12h, and 3h time intervals. In addition, leaf relative water contents and chlorophyl contents significantly reduced in cotton plants subjected to the silencing of *Ghir_D03G016230* and *Ghir_D02G013530* and subsequently treated with salt stress **Figure 8**.

## Discussion

Climate change is exacerbating the detrimental impact of high temperatures on crop growth, necessitating an intensified focus on investigating plant responses to heat stress to ensure optimal plant development (Shinozaki et al., 2006). Elevated temperatures, specifically a 5°C increase compared to normal conditions, impede physiological and biochemical reactions in organisms, leading to the inhibition of protein synthesis and mRNA transcription due to heat stress (Mittler et al., 2012). Under high-temperature stress, the expression of stress-related genes is often altered, with heat shock proteins (HSPs) being crucial for temperature acclimation. Heat shock proteins, such as HSP90, are rapidly synthesized in response to heat stress to facilitate temperature tolerance (Pearl and Prodromou, 2000; Tissiéres et al., 1974). In addition to heat stress, salt stress poses significant challenges to plant growth. Salinity, a prominent abiotic stress factor, primarily disrupts cellular homeostasis and ion distribution, leading to osmotic stress. Excessive salinity adversely affects multiple stages of plant development, causing growth retardation, biomass reduction, and leaf senescence (Munns and Tester, 2008; Parida and Das, 2005). Although heat shock protein HSP90 has been characterized in numerous plant species, limited information is available regarding HSP90 in cotton. Hence, in this study, we conducted a comprehensive analysis to identify and genetically examine the *HSP90* gene family in *Gossypium hirsutum*. Furthermore, we investigated the expression patterns of these genes in response to salt and heat stress, thereby shedding light on their potential functions in *G. hirsutum.*

In contrast to the previously identified *HSP90* gene families in other plant species, such as 7 in *A. thaliana* and 9 in rice (Krishna and Gloor, 2001), a significant increase in the number of *HSP90* genes was observed in three cotton species, with a total of 56 genes identified. This substantial expansion in gene count could be attributed to the polyploidization event that occurred in *Gossypium hirsutum* following the hybridization of *G. arboreum and G. raimondii* (Wang et al., 2018). We identified 14 *HSP90* genes each from *Gossypium arboreum* and *G. raimondii*, and 28 from *G. hirsutum*. This observation aligns with the known formation of allotetraploid *G.hirsutum* through the hybridization of *G. arboreum* and *G. raimondii* (Hu et al., 2019). The theoretical isoelectric points of all the proteins of *G.arboreum* were < 7, whereas all proteins of *G.hirsutum* were ascribed to have isoelectric points < 7 except for one protein. Only two proteins of *G.raimondii* have isoelectric point > 7. These results indicated that the HSP90 proteins of all the *Gossypium* species were acidic (Xia, 2007). Moreover, the grand average of hydropathy values of all proteins of cotton species was negative, indicating that all proteins were hydrophilic **Table 1**. The phylogenetic analysis of *Gossypium* and other plant species revealed that *HSP90* genes in subgroup III have retained higher similarities in function and structure with non-cotton species. Moreover, subgroups I and II have likely undergone more lineage-specific evolution, indicating diversification among cotton species, probably adapting to certain specific environmental conditions and functional specialization exclusive to cotton as shown in **Figure 3A**. The phylogenetic analysis of three *Gossypium* species **Figure 3B**, further corroborated the results of a combined tree of cotton and other species. the phylogenetic tree of cotton species suggested that *HSP90* genes among cotton species were not only conserved throughout evolution as subgroup III consists of genes of three cotton species showing a shared evolutionary pathway. More interestingly, it was observed that subgroup II has two genes from D subgenome of *G.hirsutum* grouped together with two genes of *G.raimondii*, which is an indication of the contribution of the special function genes from raimondii to hirsutum. These genes confirm that they have diverged from their progenitor (*G.raimondii*) to upland cotton. *G.hirsutum* is an allotetraploid species (2n= 4x=52) that originated from a hybridization event between A-genome species like *G.arboreum* or *G.herbaceum* and D genome progenitor-like *G. raimondii*. Comparative genomics analysis in previous studies has reported evidence of D-genome contribution from *G.raimondii* to *G.hirsutum.* Moreover, it can be inferred that during the inheritance of these genes there might still be some subtle evolutionary modifications that assisted *G.hirsutum* to adapt to new ecological niches or changing environmental patterns as compared to *G.raimondii*. These insights support our hypothesis that certain *HSP90* genes mediating heat tolerance also play a key role in salt tolerance. Overall, It was found that the *HSP90* genes in *Gossypium* species exhibited significant similarity and a monophyletic distribution within three phylogenetic subgroups, indicating a conserved evolutionary pattern of these genes.

In the current study, an examination of the gene structure in different groups revealed that the number of exons and the exon-intron arrangements of the *HSP90* genes within each group were highly similar as shown in **Figure 5B**. Notably, three motifs (1, 2, and 3) as depicted in **Figure 5C**, were identified as being highly conserved, which might be due to selective pressure throughout the evolutionary time. They were present in all *HSP90* genes across the three *Gossypium* species. Furthermore, the distribution of these motifs exhibited a consistent pattern among all *HSP90* genes. The highly conserved motifs often indicate the crucial functional elements within genes. These motifs play key functional roles in several important processes such as DNA binding, enzymatic actions, and protein-protein interactions. The findings of conserved motifs are indicative of their key roles necessary for the adaptations and proper functioning of the plant. Motifs which are part of the regulatory regions, may be critical for the desired expression of the genes mediating key developmental phases and stress responses. These analyses provide evidence supporting the reliability of the evolutionary classification of *HSP90* genes.

Excessive salinity, in particular, is a significant variable that adversely affects cotton production globally (Sharif et al., 2019). The analysis of *cis*-regulatory elements (CREs) revealed that the *HSP90* genes possess a substantial enrichment of important *cis*-regulatory elements that play a crucial role in mitigating environmental stresses. In the current study, a diverse array of CREs such as abscisic acid-responsive elements (ABRE), anaerobic induction, light, Methyl jasmonate (MeJA), gibberellin, drought, defense, salicylic acid, auxin, low-temperature, wound responsive CREs were found across the three cotton species **Figure 6**. Among these *cis*-regulatory elements, several CREs play pertinent roles in the salt and heat stress tolerance of cotton plants. Among these CREs, ABREs and MeJA-responsive elements play a key role in salt tolerance in plants. Previous studies have reported that the ABA hormone accumulates under salt stress and binds to the receptors initiating the ABA signaling pathway (Feng et al., 2019). This binding results in the activation of basic leucine zipper bZIP transcription factors called ABRE binding factors ABFs (Shrestha et al., 2021). The ABFs resultantly bind to the ABREs present in the promoter region of their respective genes, thereby inducing their transcription (Bhat et al., 2024). Several ABFs encode proteins crucial for the salt tolerance mechanisms such as biosynthesis of proline and glycine betaine osmolytes to maintain osmotic balance. Moreover, activation of the antioxidant system and signaling of stress-responsive signaling cascades aid in the salt tolerance of plants. Similarly, MeJA enhances salt tolerance of plants in several ways such as regulating antioxidant systems and phytohormones levels, inducing stress-responsive genes thereby protecting cell organelles and interacting with other signaling pathways (Delgado et al., 2021; Yin et al., 2023; Zhang et al., 2023). The MeJA induces the activities of reactive oxygen species scavenging enzymes such as SOD, POD, and CAT to alleviate oxidative damage caused by salt stress. Moreover, a study reported that MeJA increases the levels of auxin and cytokinins to enhance plant growth and delay senescence under salt stress (Zhang et al., 2023). These findings further support our results of comparatively elevated levels of SOD, POD, and CAT in salt-tolerant genotype HNZ2019–2520 than salt-sensitive genotype HNZ2019-252, and the potential role of MeJA responsive elements in regulating salt tolerance mechanism.

Multiple studies have provided evidence of the involvement of *HSP90* genes in response to abiotic stress (Panaretou, 1998; Pearl and Prodromou, 2000). Song et al. studied the dynamic expression levels of *Nicotiana tabacum* L. *HSP90* genes under various abiotic stress conditions, including salt, ABA, drought, cold, and heat stresses. The results demonstrated that the expression of *NtHSP90-4, NtHSP90-5*, and *NtHSP90–9* was up-regulated. Furthermore, the expression of *NtHSP90* genes was induced by salt, ABA, drought, cold, and heat stresses, indicating their potential involvement in abiotic stress responses (Song et al., 2019).

The heatmap of publicly available data of *HSP90* genes exhibited upregulation of most of the genes under heat and salt stress at different time intervals, which is indicative of the fact that *HSP90* genes play a key role in salt and heat stress tolerance. Moreover, the qRT-PCR analysis of key candidate genes exhibited differential expression of the *HSP90* genes in salt-tolerant and sensitive genotypes, which is an indication of the activity of *HSP90* genes in the salt tolerance mechanism. A previous study of *HSP90* genes in *Brassica napus* L. confirmed that *HSP90* genes play a critical role in biotic and abiotic stress, particularly under salt stress (Wang et al., 2022). Zhang et al. reported that expression of *HSP90* genes was prolonged or increased under salt stress in *Brachypodium distachyon* L (Zhang et al., 2017).

In the present study, an analysis of expression patterns revealed that the expressions of G*hir_D08G003210, Ghir_A08G003090, Ghir_A13G013590*, and *Ghir_A12G026820* were induced by salt stress. Additionally, in the qRT-PCR analysis, *Ghir_A07G020520, Ghir_D02G013530, Ghir_D03G016230*, and *Ghir_D12G026910* exhibited significant differential expression between salt-sensitive and salt-tolerant genotypes of *G.hirsutum*. Significantly higher expression of certain *HSP90* genes and elevated accumulation of SOD, POD and CAT enzymes in salt tolerant genotype explain the level of tolerance of cotton plants under salt stress. Moreover, the silencing of candidate genes *Ghir_D02G013530, and Ghir_D03G016230* revealed that the expression of SOD, POD, and CAT was intertwined with the expression of potential candidate genes of *HSP90s*. The silencing of *Ghir_D02G013530* and *Ghir_D03G016230* significantly reduced the accumulation of ROSs scavenging enzymes under salt stress and elevated the accumulation of MDA in salt tolerant genotype. It is a clear indication of the damage caused by salt stress and no/low expression of the *HSP90* candidate genes. Moreover, reduction in leaf relative water contents and chlorophyl contents paraded that silencing of the candidate genes significantly reduced the ability of cotton seedlings to tolerate the effects caused by the salt stress. These findings provide important insights into the potential role of *HSP90* genes in the salt tolerance mechanism of cotton plants. However, further functional validation of candidate genes and the production pattern of SOD, POD, CAT, and MDA in a large collection of germplasm could help to understand the markers of salt tolerance mechanism in cotton plants. In agreement with the previous reports of the potential role of *HSP90* genes in heat stress tolerance, the results of the current study revealed that *HSP90* genes not only play a key role in heat tolerance but also the salt tolerance mechanism of plants due to shared genes of multiple stress tolerance pathways.

## Data Availability

The original contributions presented in the study are included in the article/**Supplementary Material**. Further inquiries can be directed to the corresponding author/s.
