# Supplementary material for: Genome-wide identification and characterization of HSP90 family gene in cotton and their potential role in salt stress tolerance
Source: Front Plant Sci. 2025 Jul 2;16:1574604. doi: 10.3389/fpls.2025.1574604 (PMC12263452; doi:10.3389/fpls.2025.1574604)
Supplement: Supplementary file 1 [file DataSheet1.docx]

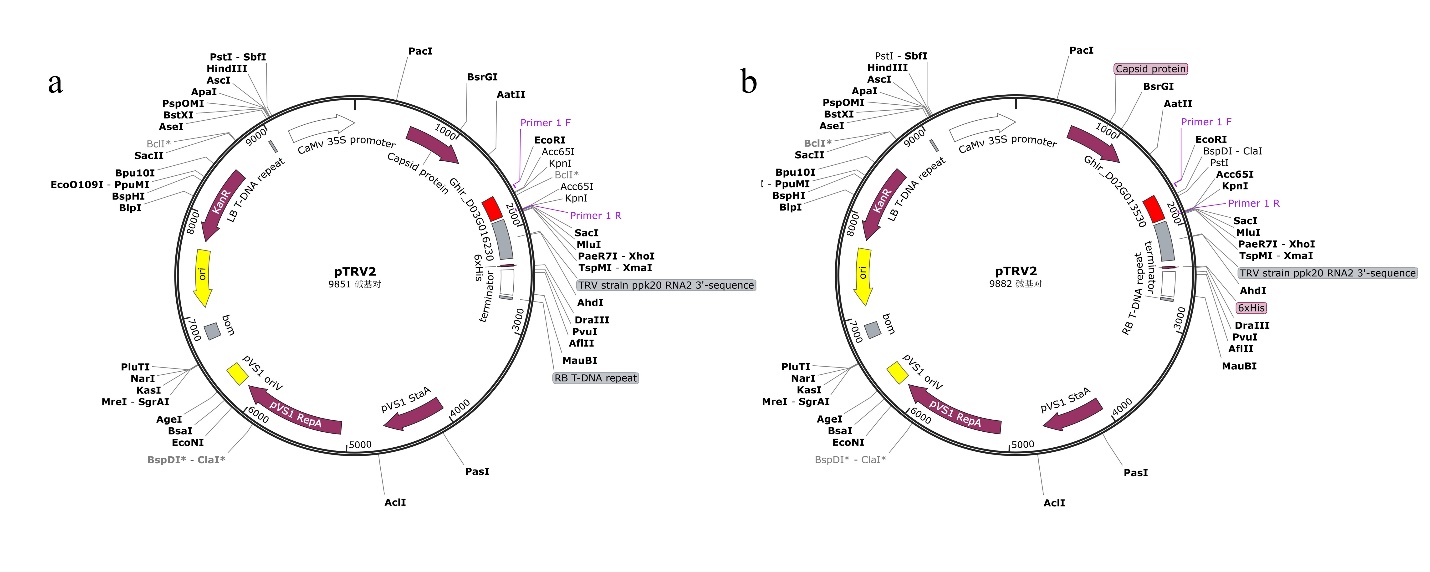


**Supplementary Figure 1: Plasmid map of TRV2. (a) The integration of** *Ghir_D03G016230* and (b) integration of *Ghir_D02G013530* in TRV2 vector.


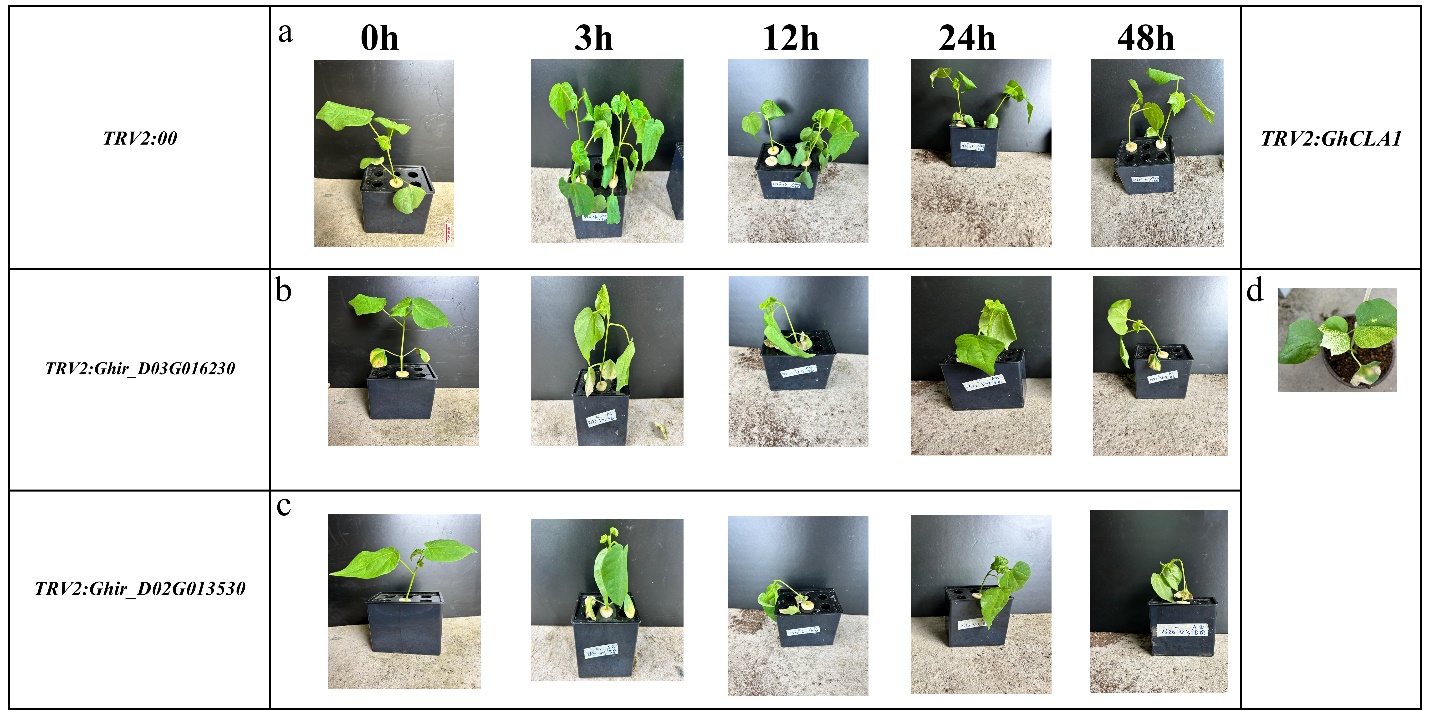
 **Supplementary Figure 1:** The phenotypic effect of *Ghir_D03G016230* and *Ghir_D02G013530* silencing on seedlings of drought tolerant cultivar under salt stress. (a) control (b) phenotypic effect of silencing *Ghir_D03G016230* (d) phenotypic effect of silencing *D02G013530* (d) The efficiency of gene silencing was assessed by detecting the albino phenotype in the newly emerging leaves in cotton seedlings treated with *TRV2:GhCLA1*
