# Supplementary material for: Genome-wide identification and characterization of HSP90 family gene in cotton and their potential role in salt stress tolerance
Source: Front Plant Sci. 2025 Jul 2;16:1574604. doi: 10.3389/fpls.2025.1574604 (PMC12263452; doi:10.3389/fpls.2025.1574604)
Supplement: Supplementary file 2 [file Table1.docx]

**Supplementary tables**

**Table S1**. The list of primers for qRT-PCR

| *Gene* | Forward primer | Reverse primer |
| --- | --- | --- |
| *Ghir_A03G002790* | CTGGTGGGTCTTTCACTGTTAC | TATCCTCCACATCACCTTCGTC |
| *Gh_A08G132800* | GAGTCTCAGGCTGGTGGGTCTT | TTGTAATCTCCTTCGGCTTTCT |
| *Ghir_A12G026820* | TGGTGGATGCCATTGATGAGTA | GTGACCAGACAGCAAGGTGAGT |
| *Ghir_D03G016230* | CTGGTGGGTCTTTCACTGTTAC | CTTCATCATCACTGACCTCCTT |
| *Ghir_D08G012920* | GGACGGCCAATATGGAGAGG | TGTGTTGGGATCATCCAGGC |
| *Ghir_D12G026910* | TGGTGGATGCCATTGATGAGTA | GTGACCAGACAGCAAGGTGAGT |
| *Ghir_A03G012060* | TTGCCATTCAGAACCTCAGACC | CTCCCAGTTGTTGCTTTATCCA |
| *Ghir_D02G013530* | AAGTGCCAAGACTGCTCCATT | CCTTGTCTCCCAGTTGTTGCT |
| *Ghir_A07G020520* | CCAGCACAGCTGAGTGGTAA | CTGTTTGGGGAGGCTGTGAT |
| *Ghir_A01G008350* | AGGGATAACAGTATGGCAGGAT | CAAAGAGGAGCAGAACAAGGTC |
| *UBQ* | GAAGGCTTCCACCTGACCAAC | CTTGACCTTCTTCTTCTTGTGCTTG |

UBQ denotes the ubiquitous/housekeeping gene

**Table S2.** The list of primers for VIGS

| # | Forward primer | Reverse primer |
| --- | --- | --- |
| connect primer | gtgagtaaggttaccgaattc | gagacgcgtgagctcggtacc |
| Ghir_D03G016230 | gtgagtaaggttaccgaattcGCTGGTGGGTCTTTCACTGTTAC | gagacgcgtgagctcggtaccTCTTCATCATCACTGACCTCCTT |
| Ghir_D02G013530 | gtgagtaaggttaccgaattcAAAGTGCCAAGACTGCTCCATT | gagacgcgtgagctcggtaccCCTTGTCTCCCAGTTGTTGCTT |

The small letters denotes the vector sequence\

**Table S3.** The Ka, Ks and Ka/Ks values of non-redundant genes and their divergence time

| **Gene1** | **Gene2** | **Ka** | **Ks** | **Ka/Ks** | **Divergence time (Mya)** |
| --- | --- | --- | --- | --- | --- |
| Ga01G0999 | Ghir_A01G008350 | 0 | 0.004672912 | 0 | 0.38 |
| Ga01G0999 | Ghir_A13G013590 | 0.013312169 | 0.811266606 | 0.016 | 66.5 |
| Ga01G0999 | Ghir_D01G008810 | 0.00120042 | 0.035756523 | 0.034 | 2.93 |
| Ga01G0999 | Ghir_D13G014300 | 0.013013541 | 0.839804876 | 0.015 | 68.84 |
| Ga01G0999 | Gorai.002G103000 | 0.002402284 | 0.030913827 | 0.078 | 2.53 |
| Ga01G0999 | Gorai.013G150300 | 0.013013541 | 0.854176574 | 0.015 | 70.01 |
| Ga01G1189 | Ghir_A01G009870 | 0.002677977 | 0.003919656 | 0.683 | 0.32 |
| Ga01G1189 | Ghir_A06G000260 | 0.048326772 | 0.399392207 | 0.121 | 32.74 |
| Ga01G1189 | Ghir_D01G010630 | 0.005268957 | 0.02927677 | 0.18 | 2.4 |
| Ga01G1189 | Ghir_D06G000070 | 0.047852528 | 0.385902604 | 0.124 | 31.63 |
| Ga01G1189 | Gorai.002G122800 | 0.005268263 | 0.019399724 | 0.272 | 1.59 |
| Ga01G1189 | Gorai.010G003000 | 0.047291255 | 0.38582095 | 0.123 | 31.62 |
| Ga01G2599 | Ghir_D03G016230 | 0.004166057 | 0.077223064 | 0.054 | 6.33 |
| Ga01G2599 | Ghir_D08G012920 | 0.025796595 | 0.862791419 | 0.03 | 70.72 |
| Ga01G2599 | Gorai.003G155600 | 0.002973834 | 0.077176462 | 0.039 | 6.33 |
| Ga01G2599 | Gorai.004G138600 | 0.025179311 | 0.870073029 | 0.029 | 71.32 |
| Ga02G1769 | Ghir_A08G003090 | 0.002405414 | 0.142529333 | 0.017 | 11.68 |
| Ga02G1769 | Ghir_A13G013590 | 0.010576324 | 0.979510147 | 0.011 | 80.29 |
| Ga02G1769 | Gorai.004G033900 | 0.003612651 | 0.093525349 | 0.039 | 7.67 |
| Ga03G1468 | Ghir_A03G012060 | 0.004813218 | 0.018242477 | 0.264 | 1.5 |
| Ga03G1468 | Ghir_D02G013530 | 0.002704045 | 0.029555622 | 0.091 | 2.42 |
| Ga03G1468 | Ghir_D13G009090 | 0.060106519 | 0.387631301 | 0.155 | 31.77 |
| Ga03G1468 | Gorai.005G148100 | 0.001080984 | 0.033525134 | 0.032 | 2.75 |
| Ga03G1468 | Gorai.013G098300 | 0.067969202 | 0.426674714 | 0.159 | 34.97 |
| Ga05G1968 | Ghir_A01G009870 | 0.196475537 | 0.925573256 | 0.212 | 75.87 |
| Ga05G1968 | Ghir_A05G018960 | 0.098556743 | 0.12040209 | 0.819 | 9.87 |
| Ga05G1968 | Ghir_A06G000260 | 0.187055433 | 1.726250431 | 0.108 | 141.5 |
| Ga05G1968 | Ghir_D01G010630 | 0.196628507 | 0.921060323 | 0.213 | 75.5 |
| Ga05G1968 | Ghir_D06G000070 | 0.184047938 | 1.13848937 | 0.162 | 93.32 |
| Ga05G1968 | Gorai.002G122800 | 0.202115862 | 0.867864881 | 0.233 | 71.14 |
| Ga05G1968 | Gorai.010G003000 | 0.184047938 | 1.13848937 | 0.162 | 93.32 |
| Ga06G0030 | Ghir_A01G009870 | 0.048274798 | 0.398123128 | 0.121 | 32.63 |
| Ga06G0030 | Ghir_A06G000260 | 0.009459585 | 0.035455623 | 0.267 | 2.91 |
| Ga06G0030 | Ghir_D01G010630 | 0.046323071 | 0.433342896 | 0.107 | 35.52 |
| Ga06G0030 | Ghir_D06G000070 | 0.011092275 | 0.069431664 | 0.16 | 5.69 |
| Ga06G0030 | Gorai.002G122800 | 0.046604473 | 0.407566947 | 0.114 | 33.41 |
| Ga06G0030 | Gorai.010G003000 | 0.010547841 | 0.071593974 | 0.147 | 5.87 |
| Ga07G2203 | Ghir_A07G020520 | 0 | 0.002458011 | 0 | 0.2 |
| Ga07G2203 | Ghir_D07G020620 | 0.003246758 | 0.028520547 | 0.114 | 2.34 |
| Ga07G2203 | Gorai.001G220600 | 0.001619725 | 0.028475423 | 0.057 | 2.33 |
| Ga08G1356 | Ghir_D03G016230 | 0.030730556 | 0.852878462 | 0.036 | 69.91 |
| Ga08G1356 | Ghir_D08G012920 | 0.00857546 | 0.06124601 | 0.14 | 5.02 |
| Ga08G1356 | Gorai.003G155600 | 0.029468769 | 0.851940801 | 0.035 | 69.83 |
| Ga08G1356 | Gorai.004G138600 | 0.007959664 | 0.058697696 | 0.136 | 4.81 |
| Ga12G0240 | Ghir_A12G026820 | 0.002392585 | 0.004617175 | 0.518 | 0.38 |
| Ga12G0240 | Ghir_D12G026910 | 0.035883957 | 0.103976807 | 0.345 | 8.52 |
| Ga12G0240 | Gorai.008G274600 | 0.003591029 | 0.075032288 | 0.048 | 6.15 |
| Ga13G1166 | Ghir_A13G009660 | 0.004482643 | 0.005928885 | 0.756 | 0.49 |
| Ga13G1166 | Ghir_D13G009090 | 0.031372044 | 0.04238895 | 0.74 | 3.47 |
| Ga13G1166 | Gorai.013G098300 | 0.036390247 | 0.088602639 | 0.411 | 7.26 |
| Ga13G1641 | Ghir_A01G008350 | 0.014535632 | 0.817588522 | 0.018 | 67.02 |
| Ga13G1641 | Ghir_D01G008810 | 0.014537833 | 0.823668631 | 0.018 | 67.51 |
| Ga13G1641 | Ghir_D13G014300 | 0.003610115 | 0.023568318 | 0.153 | 1.93 |
| Ga13G1641 | Gorai.002G103000 | 0.015758951 | 0.817875753 | 0.019 | 67.04 |
| Ga13G1641 | Gorai.013G150300 | 0.003610115 | 0.023568318 | 0.153 | 1.93 |
| Gorai.001G220600 | Ghir_A07G020520 | 6.86E-04 | 0.035197074 | 0.019 | 2.89 |
| Gorai.002G103000 | Ghir_A01G008350 | 0.002402284 | 0.035784963 | 0.067 | 2.93 |
| Gorai.002G103000 | Ghir_A13G013590 | 0.014533431 | 0.818450881 | 0.018 | 67.09 |
| Gorai.002G103000 | Ghir_D01G008810 | 0.00120036 | 0.014101473 | 0.085 | 1.16 |
| Gorai.002G103000 | Ghir_D13G014300 | 0.014234943 | 0.847260343 | 0.017 | 69.45 |
| Gorai.002G122800 | Ghir_A01G009870 | 0.005930216 | 0.021852233 | 0.271 | 1.79 |
| Gorai.002G122800 | Ghir_A05G018960 | 0.266292095 | 0.933449743 | 0.285 | 76.51 |
| Gorai.002G122800 | Ghir_A06G000260 | 0.044860916 | 0.412299667 | 0.109 | 33.8 |
| Gorai.002G122800 | Ghir_D01G010630 | 0.001051295 | 0.023291537 | 0.045 | 1.91 |
| Gorai.002G122800 | Ghir_D06G000070 | 0.045232735 | 0.396897343 | 0.114 | 32.53 |
| Gorai.003G155600 | Ghir_A03G002790 | 0.037199067 | 0.110131241 | 0.338 | 9.03 |
| Gorai.003G155600 | Ghir_D03G016230 | 5.94E-04 | 0.006903213 | 0.086 | 0.57 |
| Gorai.003G155600 | Ghir_D08G012920 | 0.025186953 | 0.839584782 | 0.03 | 68.82 |
| Gorai.004G033900 | Ghir_A08G003090 | 0.003612833 | 0.093506146 | 0.039 | 7.66 |
| Gorai.004G033900 | Ghir_A13G013590 | 0.012716018 | 0.914363359 | 0.014 | 74.95 |
| Gorai.004G033900 | Ghir_D08G003190 | 0.003010843 | 0.055092597 | 0.055 | 4.52 |
| Gorai.004G138600 | Ghir_A03G002790 | 0.052670754 | 0.661145701 | 0.08 | 54.19 |
| Gorai.004G138600 | Ghir_D03G016230 | 0.025800511 | 0.847535081 | 0.03 | 69.47 |
| Gorai.004G138600 | Ghir_D08G012920 | 0.001794796 | 0.002283976 | 0.786 | 0.19 |
| Gorai.005G148100 | Ghir_A03G012060 | 0.006469117 | 0.050926817 | 0.127 | 4.17 |
| Gorai.005G148100 | Ghir_D02G013530 | 0.001621549 | 0.023535188 | 0.069 | 1.93 |
| Gorai.005G148100 | Ghir_D13G009090 | 0.058911527 | 0.380668595 | 0.155 | 31.2 |
| Gorai.007G238900 | Ghir_D11G023100 | 0.017799121 | 0.059733047 | 0.298 | 4.9 |
| Gorai.008G274600 | Ghir_A12G026820 | 0.004792827 | 0.069902023 | 0.069 | 5.73 |
| Gorai.008G274600 | Ghir_D12G026910 | 0.033946249 | 0.039132296 | 0.867 | 3.21 |
| Gorai.010G003000 | Ghir_A01G009870 | 0.045728447 | 0.392862859 | 0.116 | 32.2 |
| Gorai.010G003000 | Ghir_A05G018960 | 0.231924063 | 1.177825044 | 0.197 | 96.54 |
| Gorai.010G003000 | Ghir_A06G000260 | 0.006352886 | 0.056430838 | 0.113 | 4.63 |
| Gorai.010G003000 | Ghir_D01G010630 | 0.044677088 | 0.413245631 | 0.108 | 33.87 |
| Gorai.010G003000 | Ghir_D06G000070 | 0.001583044 | 0.005848933 | 0.271 | 0.48 |
| Gorai.010G173700 | Ghir_D06G016230 | 0.015019275 | 0.007177088 | 2.093 | 0.59 |
| Gorai.013G098300 | Ghir_A03G012060 | 0.073231239 | 0.440438969 | 0.166 | 36.1 |
| Gorai.013G098300 | Ghir_A13G009660 | 0.037157784 | 0.091313503 | 0.407 | 7.48 |
| Gorai.013G098300 | Ghir_D02G013530 | 0.068849715 | 0.429101358 | 0.16 | 35.17 |
| Gorai.013G098300 | Ghir_D13G009090 | 0.04814882 | 0.074831749 | 0.643 | 6.13 |
| Gorai.013G150300 | Ghir_A01G008350 | 0.013013541 | 0.861467032 | 0.015 | 70.61 |
| Gorai.013G150300 | Ghir_A13G013590 | 0.003609753 | 0.02357758 | 0.153 | 1.93 |
| Gorai.013G150300 | Ghir_D01G008810 | 0.013015511 | 0.867878573 | 0.015 | 71.14 |
| Gorai.013G150300 | Ghir_D13G014300 | 0 | 0.004647575 | 0 | 0.38 |

**Table S4.** In-silico analysis of HSP90 proteins sub-cellular localization.

| **Gene ID** | **Cell-Ploc** | **WoLF PSORT** | **LocTree3** |
| --- | --- | --- | --- |
| *Ghir_A01G008350* | Cy | Cy | Cy |
| *Ghir_A01G009870* | Er/Mi | Er | Er |
| *Ghir_A03G002790* | Cy | Cy | Cy |
| *Ghir_A03G002800* | Cy | Cy | Cy |
| *Ghir_A03G012060* | Er | Ch | Cy |
| *Ghir_A05G018960* | Nu | Ch | Er |
| *Ghir_A06G000260* | Er/Mi | Er | Er |
| *Ghir_A07G020520* | Er | Cy | Cy |
| *Ghir_A08G003090* | Cy | Cy | Cy |
| *Ghir_A08G003100* | Cy | Cy | Cy |
| *Ghir_A08G026410* | Cy | Cy | Cy |
| *Ghir_A12G026820* | Cy | Cy | Cy |
| *Ghir_A13G009660* | Er | Ch | Cy |
| *Ghir_A13G013590* | Cy | Cy | Cy |
| *Ghir_D01G008810* | Cy | Cy | Cy |
| *Ghir_D01G010630* | Er/Mi | Er | Er |
| *Ghir_D02G013530* | Er | Ch | Cy |
| *Ghir_D03G016230* | Er | Cy | Cy |
| *Ghir_D06G000070* | Er/Mi | Er | Er |
| *Ghir_D06G016230* | Nu | Cy/Nu | Er |
| *Ghir_D07G020620* | Ch | Ch | Cy |
| *Ghir_D08G003190* | Cy | Cy | Cy |
| *Ghir_D08G003210* | Cy | Cy | Cy |
| *Ghir_D08G012920* | Cy | Cy | Cy |
| *Ghir_D11G023100* | / | Ch | Cy |
| *Ghir_D12G026910* | Cy | Pe | Cy |
| *Ghir_D13G009090* | Er | Ch | Cy |
| *Ghir_D13G014300* | Cy | Cy | Cy |
| *Ga01G0999* | Cy | Cy | Cy |
| *Ga01G1189* | Er/Mi | Ch | Er |
| *Ga01G2599* | Cy | Cy | Cy |
| *Ga02G1769* | Cy | Cy | Cy |
| *Ga02G1770* | Cy | Cy | Cy |
| *Ga03G1468* | Er | Ch | Cy |
| *Ga05G1968* | Nu | Ch | Er |
| *Ga06G0030* | Er/Mi | Er | Er |
| *Ga06G0031* | Er/Mi | Er | Er |
| *Ga07G2203* | Er | Mi | Cy |
| *Ga08G1356* | Cy | Cy | Cy |
| *Ga12G0240* | Cy | Cy | Cy |
| *Ga13G1166* | Er | Ch | Cy |
| *Ga13G1641* | Cy | Cy | Cy |
| *Gorai.001G220600* | Er | Mi | Cy |
| *Gorai.002G103000* | Cy | Cy | Cy |
| *Gorai.002G122800* | Er/Mi | Er | Er |
| *Gorai.003G155600* | Er | Cy | Cy |
| *Gorai.004G033900* | Cy | Cy | Cy |
| *Gorai.004G034000* | Cy | Cy | Cy |
| *Gorai.004G138600* | Cy | Cy | Cy |
| *Gorai.005G148100* | Er | Ch | Cy |
| *Gorai.007G238900* | / | / | Cy |
| *Gorai.008G274600* | Er | Cy | Cy |
| *Gorai.010G003000* | Er/Mi | Pe | Er |
| *Gorai.010G173700* | Nu | Cy/Nu | Er |
| *Gorai.013G098300* | Er | Nu | Cy |
| *Gorai.013G150300* | Cy | Cy | Cy |

Cy: Cytoplasm, Er: Endoplasmic reticulum, Mi: Mitochondrion, Nu: Nucleus, Ch: Chloroplast, Pe: Periplasmic
